# A Very Thin MCT Film in HDVIP Achieves High Absorption

**DOI:** 10.3390/s25123701

**Published:** 2025-06-13

**Authors:** Lingwei Jiang, Changhong Sun, Xiaoning Hu, Ruijun Ding, Chun Lin

**Affiliations:** 1 National Key Laboratory of Infrared Detection Technologies, Shanghai Institute of Technical Physics, Chinese Academy of Sciences, Shanghai 200083, China; jianglingwei22@mails.ucas.ac.cn (L.J.);; 2 University of Chinese Academy of Sciences, Beijing 100049, China

**Keywords:** MCT, infrared, HDVIP, loop-hole, CPA, PhC

## Abstract

Compared to the traditional flip-chip bonded focal plane array, in high-density vertically integrated photodiode (HDVIP) focal plane technology, the thickness of the mercury cadmium telluride (MCT or Hg_1−x_Cd_x_Te) layer serves as a more critical parameter. This parameter not only influences the efficiency of photon energy absorption but also defines the pn junction area, thereby affecting the magnitude of the dark current. Furthermore, it significantly impacts the manufacturability of via-hole etching and formation processes. This paper investigated the photonic crystal resonances and coherent perfect absorption (CPA) effect of a thin MCT layer in HDVIP by using COMSOL Multiphysics^®^ 4.3b and optimized the structure of the loop-hole photodiode device. The CPA, which is formed by this structure, achieves high absorption of illumination in a very thin MCT film. It is demonstrated that an absorption rate of infrared radiation of more than 95% with a wavelength during the 8 µm–10 µm range can be achieved in Hg_1−x_Cd_x_Te (x = 0.225) with a thickness of only 1.5 µm–3 µm. The benefit of thinner MCT film is that it decreases the dark current of pn junction and reduces the technical difficulty of etching and metallization of the loop-hole photodiode.

## 1. Introduction

Hybrid infrared focal plane arrays (IRFPAs) are one of the most important sensors for infrared detection. Detectors and multiplexers are fabricated independently and hybridized together using flip-chip bonding. The detector array is typically connected to the silicon multiplex pads via pressure contacts, utilizing indium bumps. At low temperatures (77 K), the indium bumps undergo tensile stress due to differing coefficients of thermal expansion between the detector material and the ROIC [1].

**Figure 1:**
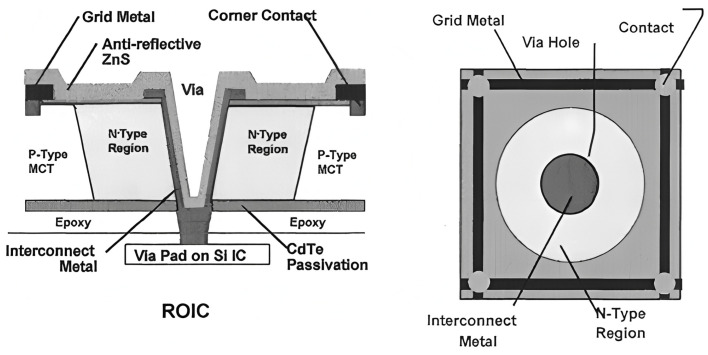
DRS HDVIP^TM^ n +/n−/p structure [2].

The HDVIP was developed at Leonardo DRS. The monocular HDVIP^TM^ structure prepared by DRS is shown in Figure 1 [2]. Detector and multiplexers are glued together, and their electrical interconnection is made through a small hole formed in each detector. DRS Network and Imaging Systems has developed ultra-small 5 µm pitch infrared detectors for the long-wave infrared (LWIR) and medium-wave infrared (MWIR) bands as part of the Defense Advanced Research Projects Agency—Alert Warefighter Enablement (DARPA AWARE) Lambda scale effort [3]. They show LWIR FPAs operating at 77 K collected 45–55% of the incident photons, and the collection efficiency could be increased by using a thicker Hg_1−x_Cd_x_Te layer [3]. However, the hole etching process limits the thickness of Hg_1−x_Cd_x_Te because of the via’s aspect ratio. Therefore, it is crucial to explore alternative methods to enhance absorption.

HDVIP^TM^ is also a two-dimensional photonic crystal slab (2D-PhC) [4]. A PhC slab can sustain optical resonances, known as guided mode resonances (GMRs) [4], which enhance transmission. CPA refers to systems that completely absorb electromagnetic radiation by controlling the interference of multiple incident waves. Wenjie Wan [5] demonstrated that coherent optical fields are completely absorbed within a resonator that contains a loss medium.

Some studies use a metal layer as the bottom reflective material to achieve Fabry–Perot interference [6,7,8]. The HDVIP structure can fulfill the conditions for such CPA by increasing the area of ROIC electrode, which functions as a reflection layer. By carefully adjusting each material thickness in HDVIP, it becomes possible to leverage photonic resonances to enhance the detector performance. Anderson [9] analyzed how photonic crystal resonances improve HDVIP’s performance, but he did not account for the effects of the metal placed beneath the epoxy layers. To date, there have been no reports indicating that both optical phenomena (2D-PhC and CPA) collectively influence absorption in HDVIP [10,11,12,13].

In this paper, we investigated the 2D-PhC and CPA phenomena in the photodiode array and optimized the HDVIP structure to achieve CPA in a very thin absorption layer with the help of COMSOL software 4.3b. Our study focused on the reflection effect of metal electrodes and an innovatively designed buried metal layer which affects electromagnetic waves’ coherence within the spectral range. To meet CPA conditions, we optimized the thicknesses of the MCT absorption layer and ZnS layer, which is the passivation material in the multilayer structure of the HDVIP. Our simulation and experiment works are mainly based on devices operating at LWIR (λ = 10.08 µm) at 77 K temperature. Theoretical calculations suggest that high absorption (>95%) can be achieved for MCT with a thickness of 1.5 µm, potentially addressing some challenges in microfabrication. The p-n junction area in HDVIP is positively correlated with the thickness of MCT; thus, thinner layers reduce dark current and simplify etching and metallization processes during photodiode preparation.

## 2. Model Design and Simulation Process

**Figure 2:**
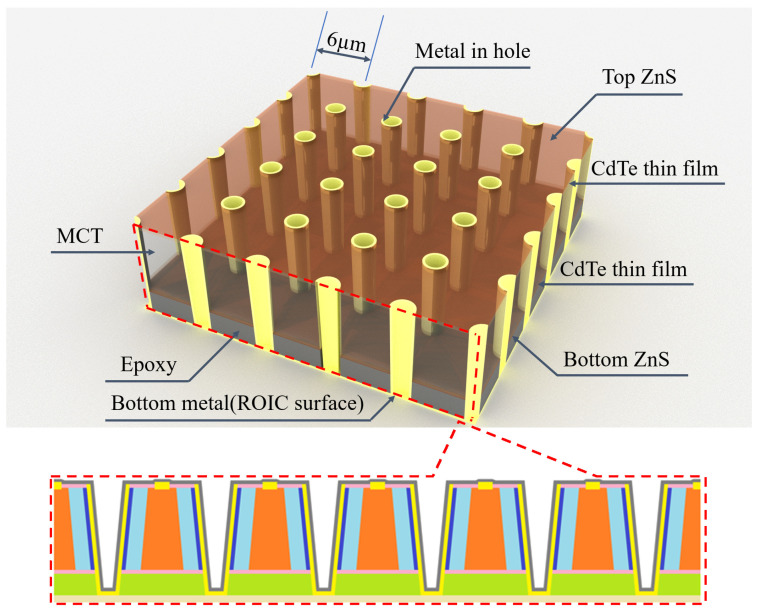
Schematic diagram of the photonic crystal structure formed by HDVIP.

The structure in Figure 1 can be viewed as a two-dimensional periodic structure in the IRFPAs. As shown in Figure 2, HDVIP is a high-density vertical hole array, which can be considered a type of photonic crystal. It has the same period as pixel pitch.

To analyze the performance of HgCdTe-based photodiodes operating at LWIR wavelengths, we set the real and imaginary parts of the refractive index (n’ and k’) to closely match those of HgCdTe with a cutoff wavelength near 10 µm at 77 K, as reported by Chu [14].

**Figure 3:**
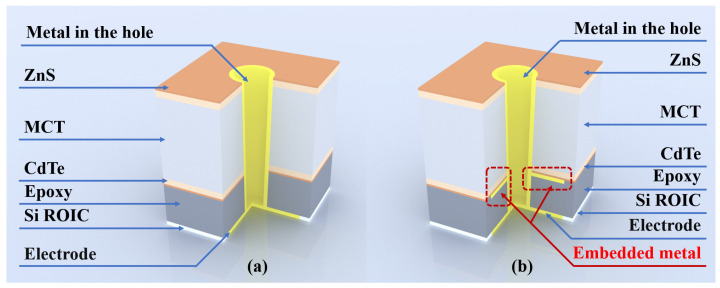
(**a**) DRS architecture and (**b**) improved architecture profile (simulation structure).

Our simulations employed single-pixel structures with Bloch boundary conditions applied in period boundaries and perfectly matched layers (PMLs) at the top and bottom. The cross-sectional architecture of a single pixel (6 µm) is illustrated in Figure 3.

In our model, we placed a metal layer on an epoxy substrate as the lower boundary, which could be achieved in the device manufacturing process. By illuminating the array with a broadband plane wave, we monitored the architecture’s reflection.

Through systematic adjustments of the HgCdTe thicknesses, ZnS layers, and via radii, resonance at specific wavelength bands can be achieved.

The material composition for x = 0.225 in long-wave Hg_1−x_Cd_x_Te results in a cutoff wavelength of 10.08 µm at 77 K [15]. The index of HgCdTe is achieved through the following formula [14]:

(1) $$n={\left(15.19-14.52x+11.06x^{2}-4.24x^{3}\right)}^{0.5},$$

(2) $$k=\frac{\alpha \lambda }{40,000\pi },$$

(3) $$\alpha =\alpha_{g}e^{{\left((\beta (E-Eg)\right)}^{0.5})},$$

(4) $$\beta =-1+0.083T+\left(21-0.13T\right)x,$$

(5) $$\alpha_{g}=-65+1.88T+\left(8694-10.31T\right)x,$$

where n is the real refractive index, k is the imaginary refractive index, α is the absorption coefficient. This paper utilized COMSOL 4.3b to compute the eigenfrequencies of this architecture.

## 3. Simulation Results and Discussion

### 3.1. Photonic Crystal Effect

**Figure 4:**
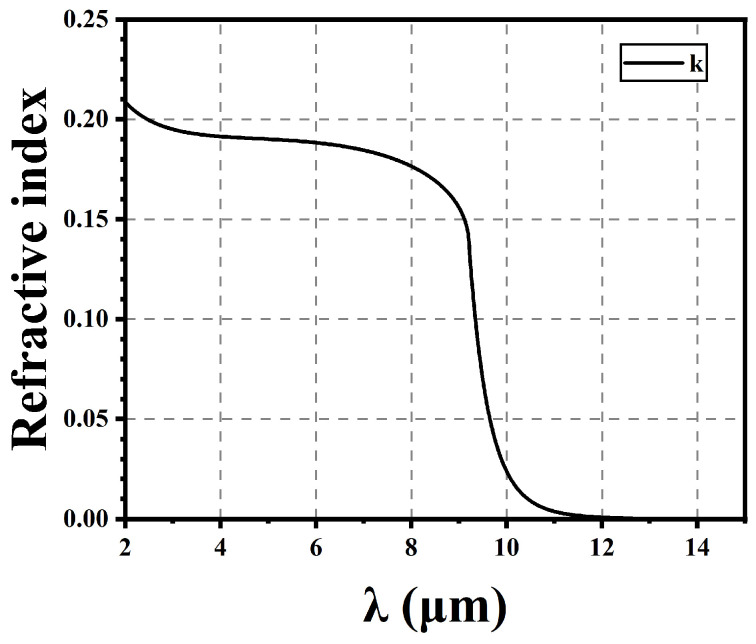
Imaginary part of the refractive index for Hg_1−x_Cd_x_Te, x = 0.225.

According to the formula in Section 2, we calculated the refractive index of Hg_1−x_Cd_x_Te (x = 0.225); the refractive index of the real part is approximately 3.52. Figure 4 shows the imaginary part of the refractive index for Hg_1−x_Cd_x_Te, and 10.08 µm is the cutoff wavelength. Actual samples will decrease in absorption more slowly at the cutoff wavelength, and some formula correction methods have struggled to achieve a very good fit [16,17,18,19].

The simulation presented in this paper is limited to the optical aspects, whereas carrier transport following photoelectric conversion has not been considered.

**Figure 5:**
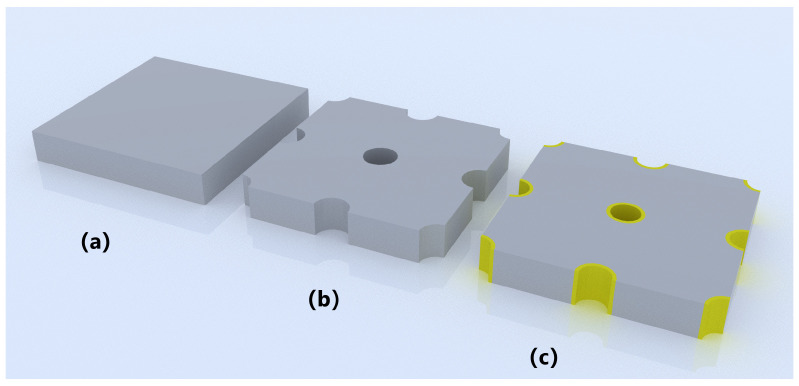
Modeling of three periodic structures. (**a**) HgCdTe single layer. (**b**) HgCdTe layer with loop-hole. (**c**) HgCdTe layer and metal in hole.

**Figure 6:**
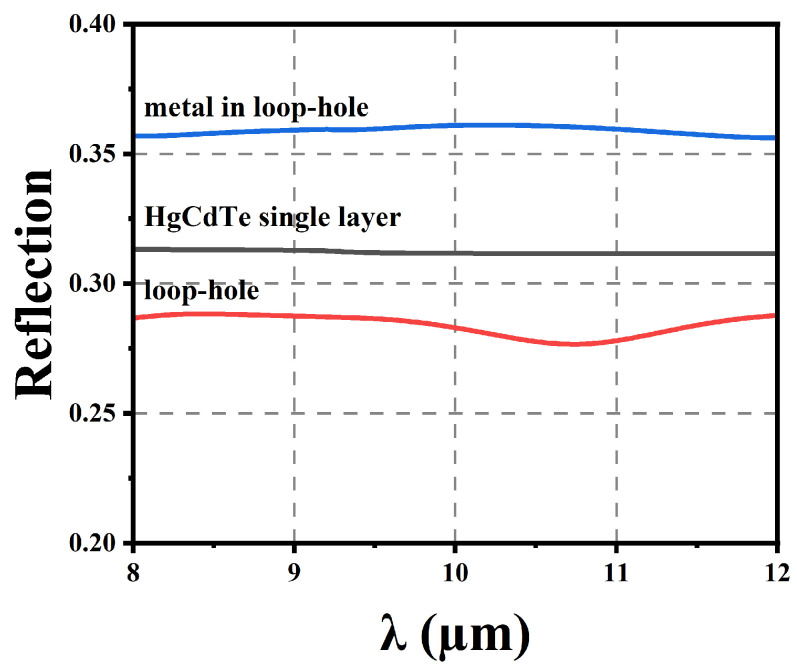
Spectroscopy of three periodic structures.

The simulation begins with an unlimited-thickness HgCdTe monolayer to determine the reflection with different structural components. Figure 5 shows the modeling of three structures. Figure 6 illustrates the reflectance of three different structural configurations. The via can influence reflection across different wavelengths.

**Figure 7:**
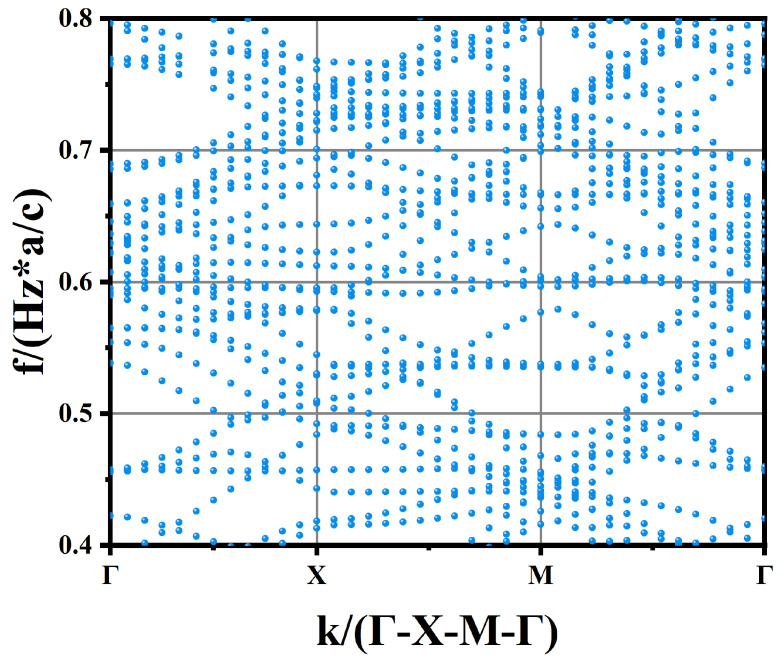
Band structure of a two-dimensional photonic crystal slab of HgCdTe.

Subsequently, a simple 2D-PhC slab model with Radius = 1.5 µm was implemented. The photonic band structure (TE mode) was computed for this configuration. The model is depicted in Figure 5b. The simulation is built by sweeping multiple k-points through the Brillouin zone of a square lattice (a = period, which is 6 µm). As shown in Figure 7, for each considered k-point, frequencies supporting guided modes for resonance (GMRs) will propagate indefinitely, while all other frequencies dissipate rapidly.

The following section describes the results of a simulation and analysis of the absorption performance, considering variations in three key parameters: the thickness of HgCdTe, the diameter of the via holes, and the thickness of ZnS.

### 3.2. Effect of the via Radius on Wave Absorption

**Figure 8:**
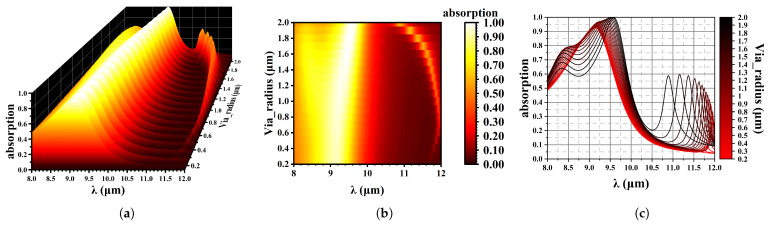
Effect of via radius on wave absorption. (**a**) 3D data mapping chart; (**b**) Contour plot, x is wavelength, y is via radius; (**c**) The absorption spectrum varies with changes in via, from 0.2 µm to 2.0 µm.

Armstrong proposed minimizing the via size for improved diode fill factor, since the via at the center of the diode does not absorb any flux [3]. However, this would greatly challenge the photolithography and etching process. This paper investigates the via radius effect on absorption. Figure 8 shows reflection spectroscopy at wavelengths ranging from 8 to 12 µm, with the following structural parameters in Table 1.

As the radius of the via increases, the position of the absorption peak remains relatively stable. However, a minor peak appears at slightly shorter wavelengths, accompanied by a shallow valley nearby. These observations suggest that photonic crystal effects are present within this structure.

**Figure 9:**
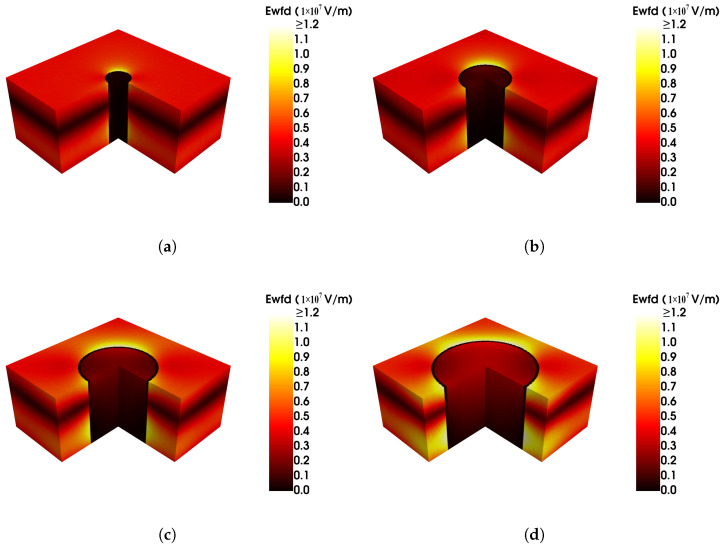
The electric field profile at a via radius of (**a**) to a radius of 0.5 µm (**b**) to a radius of 1.0 µm (**c**) to a radius of 1.5 µm (**d**) to a radius of 2.0 µm, while the wavelength of the incident wave is (**a**) 9.18 µm (**b**) 9.20 µm (**c**) 9.40 µm, and (**d**) 9.58 µm.

An increase in the via size reduces the duty cycle of the HgCdTe material. Despite this reduction, there is no significant decrease in absorption efficiency (as shown in Figure 8). This indicates that only a small fraction of the incident wave penetrates the via region; most of the incident energy is absorbed by the HgCdTe layer. This is further verified by Figure 9, which shows the electric field profile for different radii of the via ((a) 0.5 (b) 1.0 (c) 1.5 (d) 2.0 µm, with an incident wavelength of (a) 9.18 µm (b) 9.20 µm (c) 9.40 µm (d) 9.58 µm, which are all the wavelength of maximum absorption for each via). It is obvious that as the radius of the via increases, the photon density becomes higher in its peripheral region. So, differing from Armstrong’s viewpoint [3], we believe that the absorption efficiency is not proportional to the diode fill factor, and photogenerated carriers are confined to smaller regions within the architecture, which may additionally experience reduced crosstalk.

### 3.3. Effect of MCT Thickness on Wave Absorption

In general, LW HgCdTe with a thickness of 10 µm is sufficient for most photon absorption because of the MCT absorption coefficient [20]. Because the electrodes on the ROIC double the optical path via reflection and the 5 µm thick HgCdTe can receive enough photon absorption, we perform the simulation here with thinner HgCdTe, at 3 µm or less.

**Figure 10:**
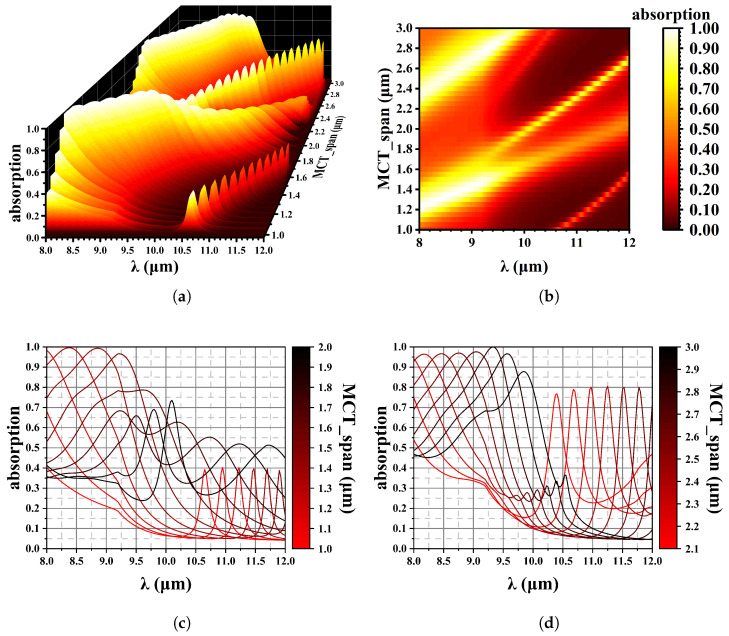
Effect of MCT thickness on wave absorption. (**a**) 3D data mapping chart; (**b**) Contour plot, x is wavelength, y is MCT thickness; (**c**) The absorption spectrum varies with changes in MCT thickness, from 1.0 µm to 2.0 µm and (**d**) from 2.1 µm to 3.0 µm.

Figure 10 illustrates reflection spectroscopy within the wavelength range of 8–12 µm. To achieve more than 95 % absorption at 8–9 µm, we can choose 1.1–1.4 µm or 2.2–2.6 µm thick HgCdTe, as shown in Figure 10b. The configuration used in this simulation is shown in Table 2. As the HgCdTe thickness increases from 1.0 µm to 3.0 µm, two distinct phenomena are observed:

The absorption peak shifts to longer wavelengths.

A secondary peak emerges at short wavelengths.

These phenomena are characterized by a periodic variation in the absorption peaks, which aligns with the CPA.

**Figure 11:**
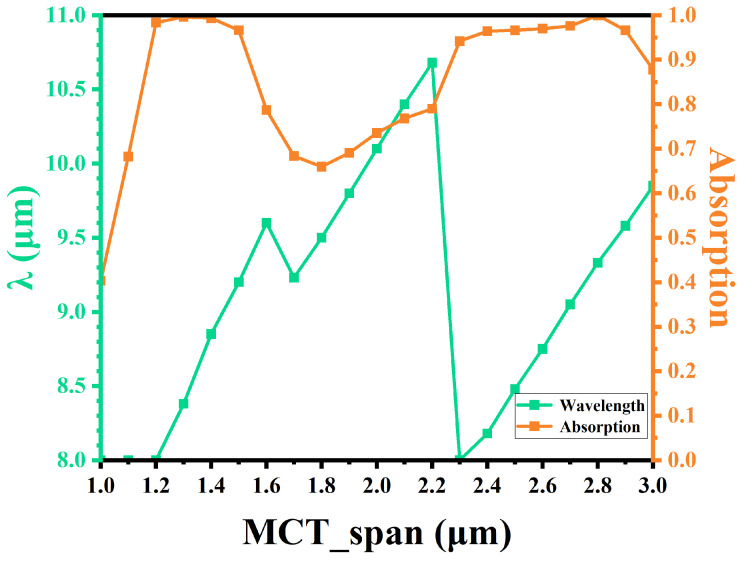
The value of the maximum absorption and the wavelength that receives the maximum absorption.

Table 3 shows the maximum absorption peak for various HgCdTe thicknesses. The absorption peak shift as a function of HgCdTe thickness is apparent. Figure 11 presents the maximum absorption value and wavelength versus HgCdTe thickness. For HgCdTe layers thinner than 1.3 µm, achieving an absorption approaching 100% becomes challenging due to the difficulty of resonance in such structures. The interference enhancement is determined by the optical path difference. Therefore, as the thickness of HgCdTe increases from 1.0 µm to 3.0 µm, the interference conditions are again satisfied in the original wavelength range.

The calculations presented in this section reveal that the thickness of HgCdTe has a significant effect on its ability to absorb the radiation.

### 3.4. Effect of the Top ZnS Thickness on Wave Absorption

**Figure 12:**
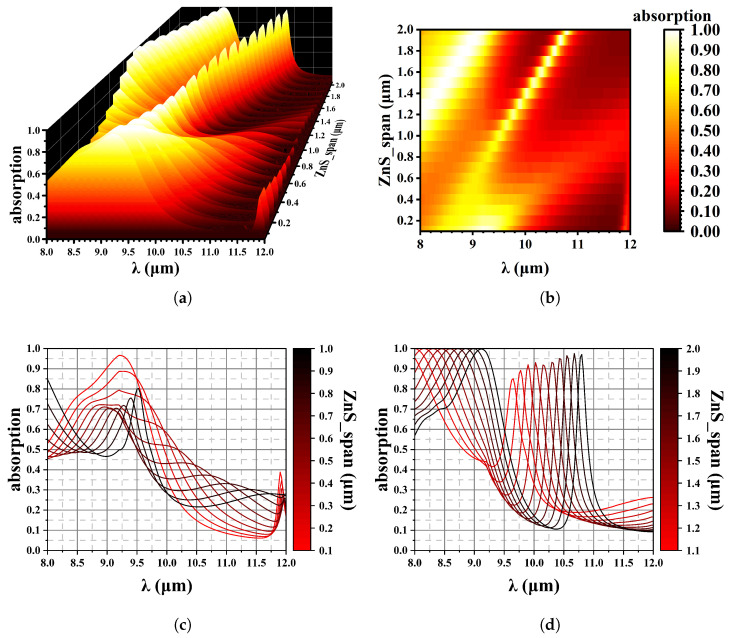
Effect of the top ZnS thickness on wave absorption. (**a**) 3D data mapping chart; (**b**) Contour plot, x is wavelength, y is ZnS thickness; (**c**) The absorption spectrum varies with changes in ZnS thickness, from 1.0 µm to 1.0 µm and (**d**) from 1.1 µm to 2.0 µm.

In the fabrication of photodetectors, the thickness of the ZnS passivation layer can be readily adjusted as a final processing step. In this study, absorption peaks are influenced by modifying the top ZnS coating while keeping all other parameters constant. As shown in Figure 12, varying the top ZnS thickness affects wave absorption when the simulation parameters are shown in Table 4.

As illustrated in Figure 12, the thickness of the ZnS layer on the surface affects the optical path of the whole material. The ZnS material is only used as a control of the peak position. Figure 12c is the spectrum of 0.1–1.0 µm thick ZnS, and Figure 12d is 1.0–2.0 µm thick ZnS. An increase in the thickness of ZnS results in a shift of the absorption peaks towards longer wavelengths, with a spectral shift of 1.41 µm/(1 µm ZnS). Consequently, by adjusting the thickness during fabrication, we can achieve the desired absorption spectra for practical applications.

The data in Table 5 show the corresponding maximum absorption peaks for different ZnS thicknesses. When the thickness increases from 1.2 µm to 2.0 µm, a significant shift of the absorption peak is observed, from 8.00 µm to 9.13 µm.

## 4. Experiments and Results

### 4.1. Test Structures

**Figure 13:**
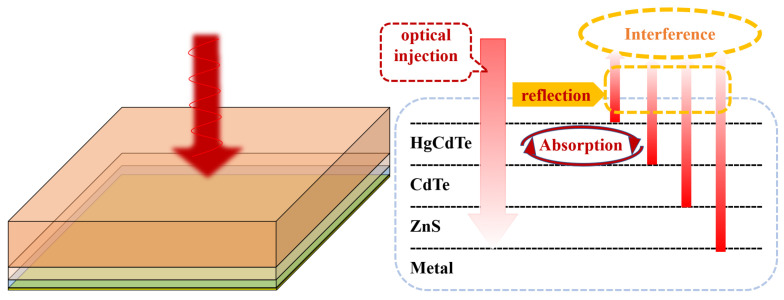
Test structure and schematic diagram.

As demonstrated by the calculations above, the effect of the periodic hole structure on the absorption of the HgCdTe material is inferior to CPA. Thus, we fabricate a test structure to verify the model simulation of the absorption of a very thin HgCdTe film, mainly considering the CPA. As shown in Figure 13, the test structure has four layers, HgCdTe/CdTe/ZnS/metal, making it relatively similar to the actual device structure. The HgCdTe layer was thinned out to 1 µm by CMP (chemical–mechanical polishing) and ICP etching. The thickness of the CdTe layer underneath the MCT is about 0.2 µm, and the ZnS underneath the CdTe is 0.1 µm. The metal is at the bottom of the structure. Therefore, there is no wave transmitted in this test structure. It can be calculated using the model established in this paper that the minimum reflection at 77 K is at a wavelength of 5.5 µm.

Since there is no electromagnetic wave transmission, the sum of the absorption and reflection of the material is 100%.

### 4.2. Measurement of Test Structures

**Figure 14:**
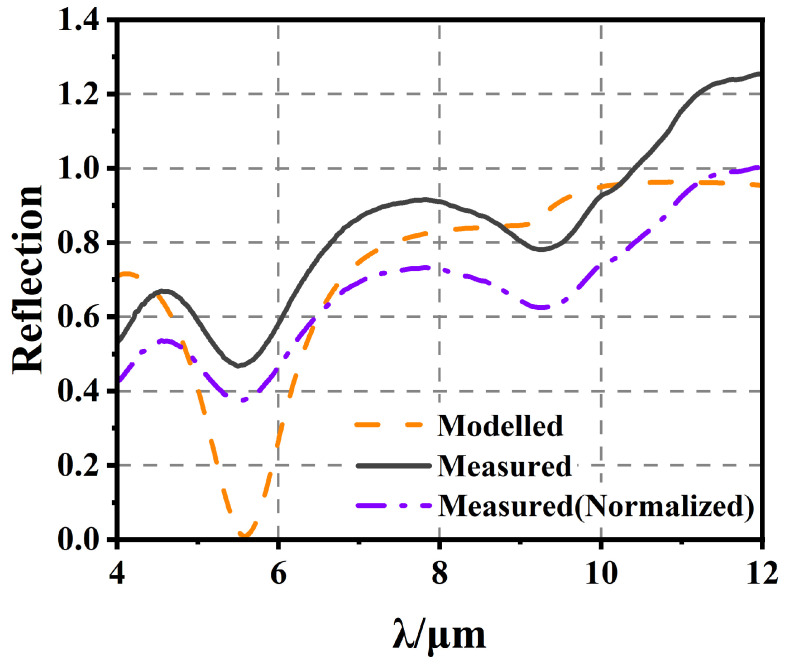
The measured FTIR and reflectance spectrum of a test structure and the modeled reflectance spectrum of the best fit structure.

The test structures were optically characterized at 77 K using FTIR. The material cutoff of the Hg_1−x_Cd_x_Te (x = 0.225) was around 10.08 µm. Figure 14 shows both the measured reflectance (before and after normalization) and best fit modeled reflectance, while the bottom layer was metal; there was no light transfer, so the wave was reflected or absorbed.

The refractive index calculation of HgCdTe showed good fitting at the cutoff wavelength. Figure 14 shows reasonable agreement from 4 to 12 µm. The background of the reflectance spectrum was compared to the Cr film’s reflection spectrum, necessitating normalization for proper fitting. The normalization process involved dividing the test data by 1.25 to position the 100% reflection after the cutoff wavelength (12 µm). After this adjustment, a better fit was achieved near 6–10 µm. This is because the material parameters of the simulation are better fitted near cutoff wavelengths. The sharp increase in the modeled reflectance at 10 µm is due to the sharp decrease in the imaginary part of the refractive index of the model after the cutoff wavelength, whereas the actual sample should be inhomogeneous in terms of the fraction of Cd, resulting in a slower change in absorptive capacity near cutoff wavelengths.

## 5. Conclusions

This paper investigates the photon absorption of the HgCdTe layer in HDVIP FPAs, presenting a simulation study aimed at achieving CPA with a very thin HgCdTe film. The infrared absorption properties of HgCdTe can be optimized by adjusting the thicknesses of the HgCdTe absorption layer and the ZnS passivation layer, as well as modifying the radius of the vias.

The dimensions of the vias have minimal influence on the absorption characteristics, indicating that optimizing other structural parameters is more critical to improving absorption. A typical structure is as follows: 1.5 µm HgCdTe, 0.2 µm CdTe, 0.1 µm bottom ZnS, 0.1 µm top ZnS, and 1.5 µm via radius. This can achieve more than 95% absorption at an 8.38 µm wavelength. The results indicate that modifying the ZnS layer’s thickness proportionally shifts the positions of peaks observed in the spectral response. HgCdTe that is more than 1.5 µm thick can reach the maximum absorption, while further increases primarily shift the position of the maximum absorption peak rather than its intensity.

Our experimental results show good agreement with the model simulation, indicating the effectiveness of the model in optimizing the diode structure to achieve CPA while considering the difficulty of the process due to the size of the deep holes and the thickness of the absorption layer.

When the thickness of HgCdTe decreases, the difficulty of the etching process and the electrode preparation process will decrease. On the other hand, the A (pn junction area) in R0A will also decrease, which has a positive effect on reducing the dark current.

## 6. Patents

Xiaoning Hu, Lingwei Jiang, Changhong Sun, Ruijun Ding—The structure of the MCT photodetector utilizing a resonant cavity for absorption: CN202421023969.6.

## Figures and Tables

**Table 1:** Simulation parameters of changing via radius.

HgCdTe/µm	ZnS/µm	CdTe/µm	Via Radius/µm
1.5	0.1	0.2	0.2
…	…	…	…
1.5	0.1	0.2	2.0

**Table 2:** Simulation parameters of changing MCT thickness.

HgCdTe/µm	ZnS/µm	CdTe/µm	Via Radius/µm
1.0	0.1	0.2	1.5
…	…	…	…
3.0	0.1	0.2	1.5

**Table 3:** The thickness of MCT and its corresponding maximum absorption wavelength and absorption rate.

HgCdTe Thickness/µm	λ/µm	Absorption
1.0	8.00	40.33%
1.1	8.00	68.22%
1.2	8.00	98.35%
1.3	8.38	99.62%
1.4	8.85	99.36%
1.5	9.60	78.68%
1.7	9.23	68.37%
1.8	9.50	65.96%
1.9	9.80	69.07%
2.0	10.10	73.53%
2.1	10.40	76.82%
2.2	10.68	79.00%
2.3	8.00	94.20%
2.4	8.18	96.43%
2.5	8.48	96.62%
2.6	8.75	96.99%
2.7	9.05	97.61%
2.8	9.33	99.96%
2.9	9.58	96.62%
3.0	9.85	87.79%

**Table 4:** Simulation parameters of changing top ZnS thickness.

HgCdTe/µm	Top ZnS/µm	Bottom ZnS/µm	CdTe/µm	Via Radius/µm
1.5	0.1	0.1	0.2	1.5
…	…	…	…	…
1.5	2.0	0.1	0.2	1.5

**Table 5:** The thickness of ZnS and its corresponding maximum absorption wavelength and absorption rate.

ZnS Thickness/µm	λ/µm	Absorption
1.2	8.00	99.98%
1.3	8.15	99.98%
1.4	8.30	99.91%
1.5	8.45	99.86%
1.6	8.60	99.86%
1.7	8.73	99.92%
1.8	8.85	99.97%
1.9	9.00	99.95%
2.0	9.13	99.75%

## Data Availability

The data presented in this study are available on request from the corresponding author.

## Abbreviations

The following abbreviations are used in this manuscript:

HDVIP	High-density Vertically Integrated Photodiode
MCT	Mercury Cadmium Telluride
IRFPAs	Infrared Focal Plane Arrays
CPA	Coherent Perfect Absorption
ROIC	Read-Out Integrated Circuit
LWIR	Long-Wave Infrared
MWIR	Medium-Wave Infrared
2D-PhC	Two-Dimensional Photonic Crystal Slab
GMRs	Guided Mode Resonances
